# Students growing up with a chronically ill family member; a survey on experienced consequences, background characteristics, and risk factors

**DOI:** 10.1186/s12889-019-7834-6

**Published:** 2019-11-08

**Authors:** Hinke M. Van der Werf, Marie Louise A. Luttik, Anneke L. Francke, Petrie F. Roodbol, Wolter Paans

**Affiliations:** 1 Research Group Nursing Diagnostics, Hanze University of Applied Sciences, Groningen Petrus Driessenstraat 3, 9714 CA Groningen, the Netherlands; 2 Nivel, Netherlands Institute for Health Services Research, Utrecht, the Netherlands; 3 APH Amsterdam Public Health research institute, Vrije Universiteit Amsterdam, Amsterdam, the Netherlands; 4 Faculty of Medical Sciences, University Medical Center Groningen, Groningen, Netherlands

**Keywords:** Chronic illness, Young caregivers, Young informal caregivers, Young adults, Students, Family

## Abstract

**Background:**

Students living with a chronically ill family member may experience significant pressure, stress, and depression due to their caregiving situation. This may also lead to them delaying or dropping out of school when the combination of being a caregiver and their education program are too demanding. This survey study aims to explore the consequences for students of bachelor or vocational education programs when they are growing up with a chronically ill family member and the influence of various background characteristics and risk factors.

**Methods:**

A survey was sent to 5997 students (aged 16–25 years) enrolled in bachelor or vocational education programs in the north of the Netherlands. The content of the survey was based on a literature study and consultation with experts. Descriptive statistics, Chi-square tests, and logistic regression analyses were performed.

**Results:**

A total of 1237 students (21%) responded to the survey. A sub group of 237 (19%) students (mean age 21(2.2); 87% female) identified themselves as growing up with a chronically ill family member. More than half (54.9%) of these students indicated that they experienced negative consequences in daily life. A significant association (OR .42, *p* < .02) was found for these consequences and the level of education for which attending vocational education yields a higher risk. In addition, growing up with a mentally ill family member was associated with a 2.74 (*p* = .04) greater risk of experiencing negative consequences in daily life compared to students living with a family member with a physical disorder or multiple disorders.

**Conclusion:**

Since a substantial number of students growing up with a chronically ill family member indicate serious physical, mental, and social consequences as a result of this care situation, awareness for this specific age-group is needed. Students with a mentally ill family member and students undertaking vocational education appear to be especially at risk. Further research is required in order to gain insight that is more in-depth into the exact type of problems that these students encounter and the specific needs that they have regarding support.

## Background

Various studies indicate that children and adolescents who are growing up with a chronically ill family member experience negative consequences from this situation in their daily lives such as mental health problems [1], problems in the parent-child relationship, and inadequate school results [2, 3]. Less is known about the specific age group of young adults between 16 and 25 years old; however, the study of Green et al. [4] investigating this specific age group also reports negative consequences in terms of higher levels of symptoms of depression and anxiety. Children, adolescents, and young adults growing up in these circumstances, therefore, may require extra attention from, e.g., school and health care professionals. Leu & Becker [5] estimate that approximately 2 to 8% of all children and adolescents in advanced industrialized societies are growing up with a chronically ill family member. Policymakers and NGO’s within the UK and Australia have substantially increased their national awareness and response to the needs of these children, though awareness is only recently emerging in other countries such as the Netherlands [5]. Previous research that was mostly conducted with young children and adolescents (age 8–16) indicated that experiencing negative consequences of growing up with a chronically ill family member generally depends on characteristics that are related to individual traits of the child but also on factors such as which family member is ill [2, 3], the type of illness of this family member [6–8], and the type of tasks that must be performed [9–12]. Earlier studies indicate that it matters which family member is ill. Barkmann et al. [13] described the influence of a physically ill parent on children and adolescents between eight and 18 years. They ascertained that these children showed both externalized (aggression and delinquency) and internalized (anxiety, depression, and social withdrawal) problems. Females growing up with a chronically ill family member especially appear to be sensitive to internalizing problems when they are experiencing puberty [13]. A meta-analysis of Sieh et al. [3] described the findings of 19 studies in children and adolescents up to 16 years of age who were growing up with a chronically ill parent. They found that behavioral problems were more prevalent in families with younger children, younger ill parents, a low average socio-economic status, and longer illness duration. Also, studies about growing up with chronically ill siblings [14] or grandparents [15] reported that children and adolescents who grow up with a chronically ill family member report outcomes that are more adverse outcomes than their peers who do not live in such a situation. The study of Pakenham & Cox [16] among 2474 students (mean age 12) is one of the first major studies comparing healthy families and those with different family members who were chronically ill. These authors found that having a chronically ill parent is associated with a greater risk of mental health difficulties compared to families with other members who were ill. While most studies primarily focus on one family member, other studies mainly focus on one type of illness. In addition, most studies are limited to children and adolescents growing up with a family member suffering from either a chronic physical or a chronic mental illness [6, 17–19] and the consequences for these youth. The study of Barkmann et al. [13] showed an elevated risk of mental health problems for adolescents if one of the parents has a mental illness. This finding is supported by the quantitative and mixed illness study of Pakenham and Cox [16]. They describe that all children growing up with a chronically ill family member report significantly more negative outcomes than children originating from a family without a member having an (chronic) illness. In addition, children growing up with parents suffering from mental illness or addiction reported more mental health difficulties compared to children growing up with a parent with a physical illness [20]. It is unclear whether this outcome also applies to children growing up with other family members suffering from a mental illness. Another variable known from several studies is the actual performance of (caregiving) tasks. Growing up with a chronically ill family member requires that children and young adults perform more tasks in their home [10–12, 21]. It is known that children and young adolescents that have to perform tasks regarding mental support or emotional care such as comforting family members and listening to problems related to the care situation experience a greater number of negative consequences in their daily life [22]. Also, the number of tasks could result in more stress and burden, however, most of this stress appears to be related to emotional situations such as watching their chronically ill family member crying, feeling stress from the other family members, and being aware of the dependency of the ill person [16, 23]. In conclusion, it can be stated that most studies on the consequences of growing up with a chronically ill family member are being performed among young children and adolescents; there is minimal research among students in the specific age group between 16 and 25 years old [9, 22]. This is inadequate since these young adults are in a critical developmental stage in which they must determine the balance between their caregiving role while simultaneously struggling with identity formation and specific developmental tasks [21, 24]. In addition, there seems to be a lack of research with heterogeneous study populations that enables determining the relative influence of various contributing to the experience of consequences in young adults growing up in this situation. The aim of the current study is twofold; 1) to explore the extent to which students in the specific age group of 16–25 years old experience negative consequences in their daily life as a result of growing up with a chronically ill family member and 2) to create insight into the relative influence of factors contributing to the experiences of these (negative) consequences.

### Research question

The main research questions are:

1. To what extent do students of bachelor or vocational education programs in the north of the Netherlands who are growing up with a chronically ill family member experience negative consequences in daily life?
2. What is the relative influence of *which member of the family is ill*, *the type of the chronic illness,* and *the types of tasks being performed* on experienced negative consequences by students?

## Method

### Design

The research questions were addressed in a cross sectional survey study.

### Study sample and recruitment

Full time students between the ages of 16 and 25 years old studying at a university of applied sciences or one of the schools for secondary vocational education in the northern part of the Netherlands were approached and asked to participate. Schools for secondary vocational education have a practical learning approach that is similar to the International Standard Classification of Education level 4 while the universities of applied sciences offer a bachelor degree similar to the International Standard Classification of Education level 6 [25]. We chose to approach students following either healthcare related studies (Nursing and Social Work) or non-health-care related studies (Law and Communication, Media, and ICT) in order to create a heterogeneous sample of students from different study programs. All students were asked to take part in a short online survey on caregiving that was sent to a total sample of 5997 students from the four selected study programs. Email addresses of the 5997 students were obtained with the help of the staff from a university of applied sciences and three schools for vocational education in order to recruit students. The email included information about the study and an ability to participate voluntarily by clicking on a web-link. There were no specific inclusion criteria; the study was introduced as a study on students growing up with a chronically ill family member. Students were asked to participate when they identified themselves as growing up with a chronically ill family member. The information about the study and a request to participate was also posted on the intranet of all of the included schools.

### Data collection and instrument

The period of data collection was 4 weeks. Two weeks and 3 weeks after the first email, a reminder was sent to students aware to reiterate the option to complete the survey. The online survey (see Additional file 1: questionnaire) consisted of 16 questions, both multiple choice and open questions about demographic characteristics (questions 1–4), which family member was ill (question 5), type of chronic illness (question 6), type of tasks performed (question 7), experienced consequences in daily life (question 8), and received support for themselves and within their family (questions 10–14). The content of the survey was based on a literature study [3, 16, 26] and consultation with ten experts in the areas of youth, nursing, or family care (nurses, psychologists, and general practitioners). Prior to the data collection, a draft survey was pilot tested for comprehensibility and feasibility among ten students growing up with a chronically ill family member. The content was discussed and adjusted in response to their feedback. Completing the survey took approximately 5 min.

### Ethical considerations

The Dutch Association for Medical Education (NVMO)-Ethical Review Board (number 865) approved the study. All students received online information by email regarding the aim and the procedures of the study prior to participation, and online informed consent was obtained before the beginning of it. Participation in this study was voluntary and students could refrain from further participation or choose not to answer certain questions without providing a reason. Data obtained from the survey was treated anonymously and stored in a secured research data centre within the Hanze University of Applied Sciences; it was only accessible by the concerned research team.

### Data analysis

The survey data were analyzed using the Statistical Package for the Social Sciences (SPSS) version 23. Descriptive statistics were employed to describe the study population. Chi-square tests were used to test the differences in categorical variables among various groups (experiencing or not experiencing negative consequences in personal life). A logistic regression analysis was performed in order to determine the association of the variables *of which family member was ill, type of chronic illness,* and *type of tasks performed* on the dependent variable *experienced consequences in daily life*. We did not use a strict cutoff for the inclusion of variables. Based on recent literature, we considered all variables to be of potential influence on experiencing consequences. The model was built through backwards elimination of the non-significant predictor variables. Age, gender, and level of education were added to the model as covariates. A level of *p*-value <.05 was considered statistically significant. The odds ratios (OR) and 95% confidence intervals (CI) were calculated in a logistic regression analysis.

## Results

### Characteristics of the participants

Of the 5997 students invited to participate, a total of 237 students (3.95%) identified themselves as growing up with a chronically ill family member and were thus included in the study.

The characteristics of this group of students are provided in Table 1. Five respondents (2.1%) were excluded due to missing information (*age*, *gender*, *study,* and *which family member was ill*). The mean age of the participating students was 21.2 (SD 2.2) years. The majority of the population comprised 207 female students (87%); 158 (66.7%) of these students were studying nursing. Furthermore, a skewed distribution was ascertained for the level of education with 158 students studying in a bachelor program of a university of applied sciences (68.1%).

**Table 1:** Characteristics of young adults growing up with a chronically ill family member (*N* = 232)

	Mean	SD
Age	21.2	2.2
	N	%
Age 16–20 yrs	93	41.4
Age 20–25 yrs	139	58.6
Gender		
Female	207	87.3
Male	25	11.7
Level of education		
Bachelor education	158	68.1
Vocational education of applied sciences	74	31.9
Study program		
Nursing	158	66.7
Social work	27	11.4
Economy /Law	19	8.0
Communication/Communication and multimedia design	28	11.7

Number of students experiencing consequences

More than half (54.9%) of the students who are growing up with a chronically ill family member experience negative consequences in daily life such as physical, school related, mental, and/or social related consequences.

### Univariable analyses

Univariable analyses were performed to explore the differences in characteristics between students who do or do not experience consequences in daily life. The variables of *age and gender* did not show significant associations as shown in Table 2. *Level of education* demonstrated a trend towards more students experiencing negative consequences in their daily life when studying at a school of vocational education (*p* = .07).

**Table 2:** Students growing up with a chronically ill family member divided by experiencing daily life consequences

Variables	Presence of experienced consequences in daily life (*N* = 125)	Absence of experienced consequences in daily life (*N* = 107)	p *
	N %	N %	
Age			.67
Age 16–20	48 (38.4%)	44 (41.1%)	
Age 21–25	77 (61.6%)	63 (58.9%)	
Gender			.52
Female	110 (88.0%)	97 (90.7%)	
Male	15 (12.0%)	10 (9.3%)	
Level of education			.07
Bachelor education	76 (60.8%)	77 (72.0%)	
Vocational education	49 (39.2%)	30 (28.0%)	
Type of family member being ill			.35
Mother	41 (32.8%)	37 (34.6%)	
Father	23 (18.4%)	26 (24.3%)	
Sibling	30 (24.0%)	26 (24.3%)	
Other^a^	8 (6.4%)	8 (7.5%)	
Multiple	23 (18.4%)	10 (9.3%)	
Type of illness			.00**
Physical disorder	46 (36.8%)	73 (68.2%)	
Mentally disorder^b^	39 (31.2%)	12 (11.2%)	
Multiple health issues	40 (32.0%)	22 (20.6%)	
Performing tasks			.27
Household chores	64 (51.2%)	67 (62.6%)	
Emotional tasks^c^	15 (12.0%)	10 (9.3%)	
Multiple	44 (35.2%)	27 (25.2%)	
No tasks	2 (1.6%)	3 (2.8%)	

*Chi-square test was used **p = < .05 ^a^ Grandparents, family in law, aunts and cousins. ^b^ Mental disorders and addiction related problems.^c^ Comforting family members and listening to problems related to the (consequences) of the chronically ill family member

### Type of family member being ill

No statistically significant difference could be found for the expectation that students growing up with a chronically ill parent would experience more consequences in their personal life compared to growing up with a chronically ill sibling (*p* = .35).

### Type of illness

A significant association was found for the type of illness and experiencing consequences in daily life. Table 2 indicates a greater risk of experiencing consequences in daily life for students who are growing up with a mentally ill family member or a family member with multiple diseases (*p* < 0.001).

### Type of tasks being performed

No statistically significant difference could be found for the expectation that students experience more consequences in daily life when they have to perform tasks regarding support such as comforting family members and listening to problems related to the consequences of the chronically ill family member instead of household chores. No significant association was found for performing tasks and experiencing consequences (*p =* .27).

### Logistic regression

The overall model was statistically significant (X2[df = 12] = 40.268 *p* < .001), and the overall percentage of correct classification was 68.5%. The results as shown in Table 3 indicate a significant association (*p* < 0.02) for *experienced negative consequences in daily life* and the *level of education;* students at the universities of applied science in this study experience fewer consequences than students of vocational education schools. In addition, growing up with a family member with a physical disorder also seems to be associated with fewer negative consequences in daily life (OR 0.41; *p* < .03) while doing so with a mentally ill family member is associated with a 2.74 greater risk of experiencing consequences in daily life.

**Table 3:** Odds ratios from the binary logistic regression predicting experienced consequences in daily life

Predictors	Adjusted OR (95% CI)	*p*
Age		
Age 16–20	.97(.48–1.96)	.93
Age 21–25	Reference	
Gender		
Male	1.33(.50–3.55)	.57
Female	Reference	
Level of education		
Bachelor education	.42(.20–.88)	.02*
Vocational education	Reference	
Type of family member being ill		
Father	.51(.17-1.49)	.22
Mother	.81 (.30–2.23)	.68
Sibling	.38 (.13–1.12)	.08
Other^a^	.44 (.11–1.82)	.26
Multiple	Reference	
Type of chronic illness		
Physical disorder	.41 (.21–.94)	.03*
Mental disorder^b^	2.74 (1.06–7.07)	.04*
Multiple health issues	Reference	
Performing tasks		
Household chores	.67 (.14–3.18)	.62
Emotional tasks^c^	1.83 (.32–10.45)	.50
Multiple	3.03 (.70–13.15)	.14
No tasks	Reference	

*p = < .05 ^a^ Grandparents, family in law, aunts and cousins. ^b^ Mental disorders and addiction related problems. ^c^ Comforting family members and listening to problems related to the (consequences) of the chronically ill family member

## Discussion

Only a few studies are known to have focused on students growing up with a chronically ill family member. The current study indicates that more than half of the students that participated (*n* = 125), aged between 16 and 25 years, growing up in this situation experience negative consequences in their daily life. Especially growing up with a mentally ill family member puts them at greater risk for this. Furthermore also students in vocational education experience more consequences compared to bachelor students. The finding that growing up with a mentally ill rather than a physically ill family member increases the risk of negative consequences is consistent with the findings of Barkmann et al. [13] and Ireland and Pakenham [20]. One proposed underlying mechanism is that prevailing stigma and a lack of open communication, primarily in families with psychological and addiction problems, present a barrier to talking freely about the family situation [27]. Furthermore, compared to physical illnesses, symptoms of mental illnesses and addiction are often less understood in the community [20, 28]. This could lead to increasing pressure on this group of students who, during this time of their life, also often struggle with their personal identity [29, 30]. The finding that students of vocational education schools experience more consequences than their peers studying at the universities of applied science could be explained with the stress and coping theory of Lazarus [31]. Lazarus states that children and adolescents from families with a lower socio-economic status may lack the resources that are necessary to cope with a chronically ill family member and experience more stress which could lead to experiencing more consequences in daily life [31]. Dearden and Becker [24] also reported a relationship between disability, illness, caregiving, and low income as a recurring theme; most families in their study were dependent on welfare benefits and experienced poverty and social exclusion. These authors described the likelihood of being a student in higher education as reduced among young adults aged 16–24 growing up with a chronically ill family member [24]. Our finding that there was no significant difference between experiencing consequences and type of family member being ill contrasts with the study of Pakenham & Cox [16]. The latter authors found significant associations between the illness of the parents students experiencing consequences in daily life (mean age 12), probably related to the fact that parents normally perform the most instrumental and emotional care tasks within families. These tasks are necessary in the development of children and young adults. Also, emotional support is instrumental and highly desirable in the period of adolescence [21, 29, 30, 32]. An explanation of our finding could be that students typically surround themselves with peers and, therefore, they may be less dependent on parental support [29, 33]. Further research is needed to investigate whether the importance and quality of this peer support is sufficient and comparable with parental support. Although most literature described negative consequences while growing up with a chronically ill family member, positive effects were also found. For example, Dearden & Becker [24] and Heyman & Heyman [34] found that young caregivers generally become more mature and learn to take on more responsibility compared with peers who do not grow up in this situation. Moreover, it is possible that these young adults do not associate their family situation with any negative consequences because they view their situation and the accompanying tasks as being normal [18, 22]. This might explain that no relationship was found between *type of tasks being performed* and experiencing negative consequences in daily life. It might be that young adults that grow up with a chronically ill family member have become socially and cognitively more advanced compared to their peers, which may result in that they, and also their family members, expect themselves to be able to perform the caregiving tasks. Further research is needed to confirm these explanations.

### Limitations

Not recognizing themselves as a young adult growing up with a chronically ill family member created challenges for recruiting students for this study and may partly explain the initial response of 3.95%. Although this percentage corresponds with the findings of Leu & Becker [5] who describe an estimation of approximately 2 to 8% of all children and adolescents growing up with a chronically ill family member, we believe that there may have been more students growing up in this situation. We received reactions from students who wondered whether they could identify themselves as growing up with a chronically ill family member as they assumed that the illness of their family member was not severe, or they believed that they did not perform any tasks. Students could have assumed that they had to perform tasks to meet the inclusion criteria of being a caregiver. Most students indeed indicated that they perform tasks, however, a small number did experience consequences in daily life without performing tasks. Although we offered a video to help students determine whether they were eligible to participate in the study, some students might not have recognized themselves as caregivers and elected to not respond to our survey. In addition, a number of students informed us that the survey confronted them with the difficult aspects of their lives. Students may have decided not to respond to avoid this confrontation. This could have led to an unintended selection of participants and, subsequently, some biased results. Students experiencing the most negative consequences in daily life may not have responded to this survey. They might concern the “hidden group” that often has less contact with school [10]. Furthermore, only a short survey, addressing a limited number of variables, was created in order to obtain a sufficient response of this hard to recruit population. The width of the confidence interval for this study indicates that the small sample size may have impacted the statistical significance. We recommend larger and comparing studies with students growing up and not growing up with a chronically ill family member which would provide a more precise picture of this specific age-group. Much more and extensive research that incorporates other important variables such as an individual student’s characteristics, marital status of parents, and intra family communication is needed to gain in-depth insight into determinants of short- and long-term impact of growing up with a chronically ill family member and into the specific needs for support of this specific group.

### Clinical and scientific implications

In this study, more than half of the students growing up with a chronically ill family member indicated that they experience negative consequences in their daily life. Growing up in such a care situation may impact the health and (social) development of these students and should therefore be taken as a serious concern for health and educational organizations. Healthcare professionals are generally working from a patient oriented perspective. However, since health care in most western societies is changing towards a greater emphasis on the involvement and support of family and informal care, there is also growing awareness of the burden on these families and informal caregivers. This study indicates that this awareness should also be created for the specifically vulnerable group of young adults growing up in a family that is dealing with serious health issues. We would recommend investigating whether discussing problems within a family also helps to reduce the problems of students who are growing up with a chronically ill family member [35]. Also, school professionals such as lecturers and especially those in vocational education need to be aware of particular behaviors of their students in order to identify those who are growing up in a care situation and refer them for support if it is necessary. Studies show that most young adults are not being recognized which results in them not receiving any support [22, 26, 36, 37]. We would recommend the development of specific guidelines for lecturers on how to recognize young adults growing up in this situation. Furthermore, easily accessible information about, e.g., psychological or financial help should be available for these lecturers in order to be able to support these students. However, we also emphasize that more research is needed to gain in-depth insight in the specific nature of the problems that these students experience and to subsequently develop and test effective types of support or interventions.

## Conclusion

Students growing up with a family member with a mental disorder appear to have a greater chance of experiencing consequences in daily life compared to other students growing up with a family member with a physical illness or disorder. In addition, this study points out that students studying a vocational education have a relatively greater chance of experiencing consequences in daily life when growing up in this care situation. Further research is needed to gain insight that is more in-depth into the exact type of problems that these young adults encounter and the specific needs that they have regarding support.

## Supplementary information

**Additional file 1.** Questionnaire The online survey consisted of 16 questions, both multiple choice and open questions about demographic characteristics, which family member was ill, type of chronic illness , type of tasks performed , experienced consequences in daily life , and received support for themselves and within their family.

## Data Availability

Data on which the conclusions of the manuscript rely are presented in the main paper. Data collected and analyzed during the current study are available from the corresponding author upon reasonable request.

## Abbreviations

NVMO Ethical Review Board: Nederlandse Vereninging voor Medisch Onderwijs, The Dutch Association for Medical Education Ethical Review Board
