# Supplementary material for: Students growing up with a chronically ill family member; a survey on experienced consequences, background characteristics, and risk factors
Source: BMC Public Health. 2019 Nov 8;19:1486. doi: 10.1186/s12889-019-7834-6 (PMC6842204; doi:10.1186/s12889-019-7834-6)
Supplement: Supplementary file 1 — Additional file 1. Questionnaire The online survey consisted of 16 questions, both multiple choice and open questions about demographic characteristics, which family member was ill, type of chronic illness , type of tasks performed , experienced consequences in daily life , and received support for themselves and within their family. [file 12889_2019_7834_MOESM1_ESM.docx]

Additional file: Questionnaire

Survey Who Cares?

1. What is your gender?

2. What is your year of birth?

3. I study at;

o University of applied sciences

o School of secondary vocational education

4. I study;

o Nursing

o Social Work

o Law

o Communication, Media and ICT

5. You have indicated that you are growing up or have grown up with a chronically ill family member; please indicate who this family member is (multiple answers possible)?

o Father

o Mother

o Brother (s) / Sister (s)

o Different (open question)

6. Please indicate which type of disorder is involved (multiple answers possible)?

o Physical disorder

o Mental illness

o A disability

o An addiction

o Different (open)

7. Please indicate which tasks you perform at home (multiple answers possible)?

o Household duties (for example; doing groceries, cooking, cleaning).

o Emotional tasks (for example; comforting parent(s), looking after your brother (s) and sister (s)).

o Care duties (for example; helping with supporting / washing / dressing your parent(s)/sibling(s)).

o Practical task (for example; going to the hospital, filling in forms, etc.).

o None of them

o Different (open question)

8. Do you personally experience problems and / or complaints related to growing up with your chronically ill family member?

o No (go to question 11)

o Yes

9. Which problems and / or complaints are involved (multiple answers possible)?

o Psychological complaints (eg sadness, many worries).

o Health complaints (for example; fatigue, low resistance, etc.).

o Stress (for example; school, home situation, finances etc.).

o Problems within the family (for example; stress / quarrel at home).

o Social problems with peers (allienation, little time to meet etc.).

o School performance (difficulty meeting deadlines, diminish grades, etc.).

o Different

10. Are you currently receiving support in this situation (multiple answers are possible)?

o No, I don't get any help or support.

o Yes, practical support, for example; support from family / neighbors / friends

o Yes, practical support, for example; support / advice at school

o Yes, emotional support, for example; conversations with family / neighbors / friends

o Yes, emotional support such as conversations at school

o Yes, emotional support, such as conversations with peers

o Yes, emotional support such as psychological help

o Different

11. Is there any support present within your home situation (multiple answers possible)?

o No, there is no help or support

o Yes, practical support such as home care

o Yes, practical support, such as domestic support

o Yes, practical support, for example; support from family / neighbors / friends

o Yes, emotional support, for example; conversations with family / neighbors / friends

o Yes, emotional support such as psychological support

o I do not know exactly if there is support available

o Different

12. Do you need more or different support (multiple answers are possible)?

o No, I do not need more or other support

o Yes, practical support, for example, support from family / neighbors

o Yes, practical support, such as support / advice at school

o Yes, emotional support, for example, conversations with family / neighbors / friends

o Yes, emotional support such as conversations at school

o Yes, emotional support with peers

o Yes, emotional support such as psychological support

o Different

13. Is there currently a need for more or different support within your home situation (multiple answers are possible)?

o No, there is no need for more or other support

o Yes, practical support such as home care

o Yes, practical support such as domestic help

o Yes, practical support such as support from family / neighbors / friends

o Yes, emotional support such as psychological support

o Yes, emotional support, for example, conversations with neighbors / family / friends

o I do not know exactly whether there is a need for more or other support

o Different

14. Have you been in contact with a healthcare professional in the last 2 years regarding your home situation (multiple answers are possible)?

o No, I have not been in contact with a healthcare professional

o Yes, with the district nurse and / or home care

o Yes, with a doctor

o Yes, with a psychologist

o Yes, with a member of the care desk

o Different

15. May we approach you with the question whether you want to participate in a follow-up study (in the form of interviews) about students who grow up with a (chronically) ill family member?

o Yes, you can approach me with the question if I want to participate in follow-up research.

o No, you may not approach me with the question whether I want to participate in follow-up research.

16. If you have indicated that we can approach you for a follow-up study, would you like to write your email address below?
